# Lingual orthodontics for children and adolescents: improvement of the indirect bonding protocol

**DOI:** 10.1186/1746-160X-9-27

**Published:** 2013-09-11

**Authors:** Frauke Beyling, Rainer Schwestka-Polly, Dirk Wiechmann

**Affiliations:** 1 Department of Orthodontics, Hannover Medical School, 30625 Hannover, Germany; 2 Orthodontic Practice, Lindenstrasse 44, 49152 Bad Essen, Germany

## Abstract

**Introduction:**

Demineralization of the dental enamel is a finding associated with fixed orthodontic treatment. When an indirect bonding procedure is used in children and adolescents the area beneath the bracket base may be affected.

**Aim:**

To evaluate if the addition of an extra layer of a hydrophilic resin, to a conventional indirect bonding protocol, can reduce the incidence of demineralization beneath the bracket base.

**Methods:**

40 patients under 18 years of age were treated with completely customized lingual appliances. Two different bonding protocols were used either with or without the application of an additional layer of hydrophilic resin. Demineralization beneath the bracket base, after de-bonding, was evaluated by standardized intra-oral photographs.

**Results:**

The addition of an extra layer of a hydrophilic resin helps to reduce the number of demineralized areas beneath the bracket bases significantly (three times less). The severity of the few remaining defects were minor and without any clinical consequence.

**Conclusion:**

When bonding a completely customized lingual appliance in children and adolescents, an extra layer of a hydrophilic resin should be added to the teeth.

## Introduction

**Figure 1:**
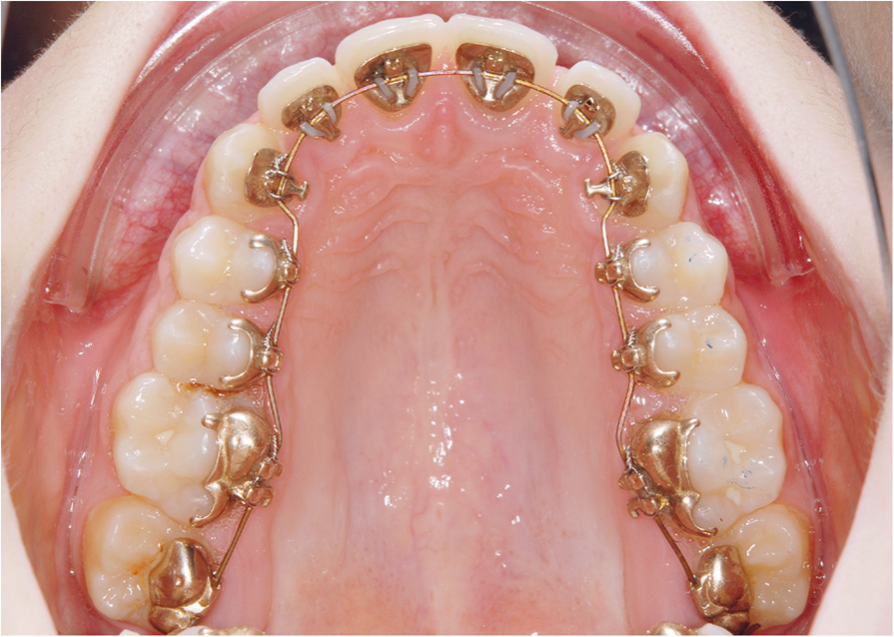
Completely customized lingual appliances (CCLA), Incognito™ (3 M Top Service für Lingualtechnik, Bad Essen, Germany).

With the introduction of completely customized lingual appliances (CCLA), a growing number of children and adolescents are now being treated with this technique [1,2] (Figure 1). Besides the aesthetic advantages of CCLA, they have also been shown to reduce the enamel decalcification risk during comprehensive orthodontic treatment.

Van der Veen et al. [3], in a prospective randomized split-mouth study, showed the incidence of white spot lesions (WSL) on the buccal surfaces around the labial brackets, to be almost five times higher than developing or progressing lesions found on the lingual surfaces with lingual brackets. Additionally, besides the number of lesions, their severity as measured by the integrated calcium loss, was on average ten times higher with labial fixed appliances than with lingual fixed appliances [3]. Knowing that WSL are a frequent and irreversible problem in relation to orthodontic treatment with fixed appliances, lingual orthodontic treatment has the potential to improve the quality of comprehensive orthodontic care [4-10].

**Figure 2:**
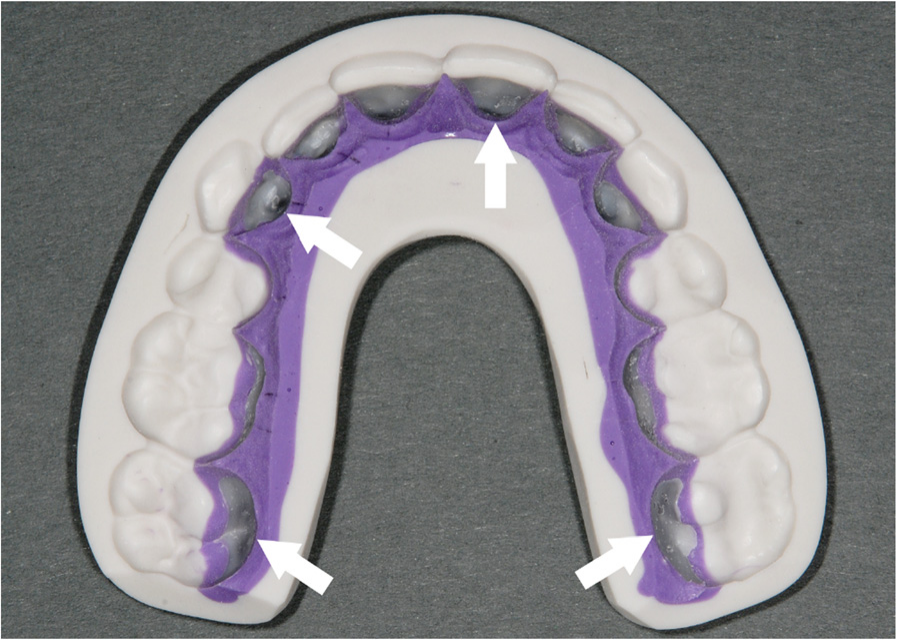
**Silicon transfer tray for the entire upper arch including the customized lingual brackets.** Arrow shows some of the lingual bracket bases with a resin coating, ready for bonding.

Because of the anatomical variations in the lingual tooth surfaces and the increased importance of correct bracket placement, particularly in the third order, direct bonding of lingual brackets has been shown to be imprecise and leads to major problems at the finishing stages of treatment [11]. The use of a laboratory procedure for customized lingual bracket placement has become a standard protocol. The placement of a lingual appliance into the mouth is carried out using an indirect bonding procedure [11-13]. In contrast to direct bonding methods, indirect bonding could involve leaving voids between the individual bracket bases and the teeth if it is carried out with a transfer tray for the entire dental arch (Figure 2). The reasons for these voids are mainly minor tooth movements that can occur between the day of the impression taking and the day of the bonding. These movements could result in minor variations of the fit of the bracket base to the tooth surface, when the bonding tray is in its final position. Although a bonding resin is applied on both the bracket base and tooth surface, the gap may not be completely filled with resin. This can lead to carious lesions under the bracket, which will be defined as sub bracket lesions (SBL).

**Figure 3:**
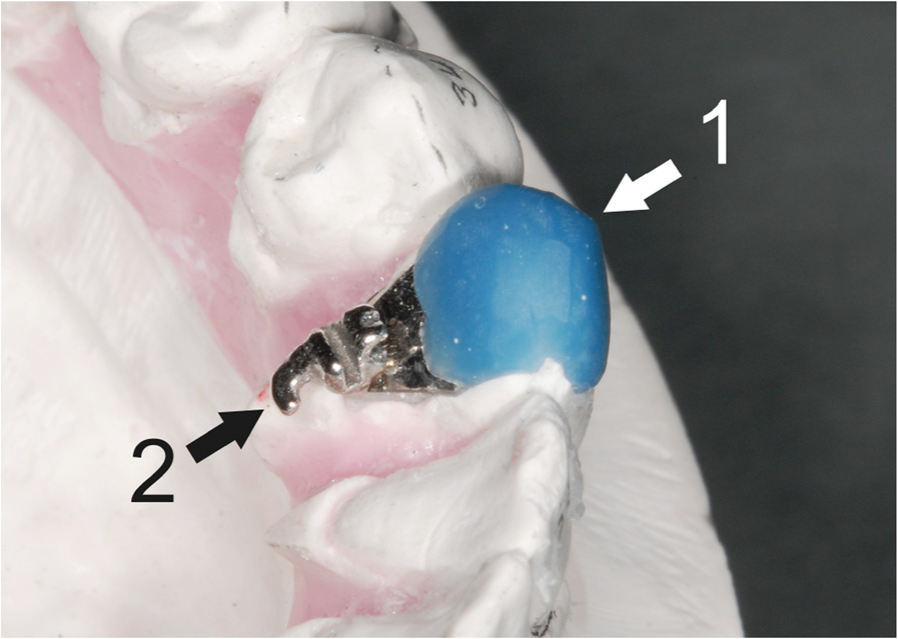
Single transfer Jig (1), on a plaster cast, with a lingual bracket (2).

Indirect bonding procedures could also be carried out with the use of single transfer jigs instead of a transfer tray, but the risk of wrong bracket placement especially in areas with crowding may compromise the quality of the final outcome (Figure 3). Furthermore not only for the practitioner but also for young patients, it is desirable to keep the bonding appointment short; therefore bonding with a transfer tray is recommended [11-13] (Figure 2).

This study investigated if the addition of an extra layer of bonding resin helped to prevent voids between the bracket base and the tooth surface, reducing the development of SBL in lingual orthodontic treatment of children and adolescents.

### Subjects and methods

#### Subjects

The sample of this study consisted initially of 45 patients treated in a private orthodontic clinic (Wiechmann and Partners, Bad Essen, Germany) from 2010 to 2012. Following the introduction of a new modified bonding protocol in the practice in April 2010, a group of 20 consecutively bonded patients, who had received this new bonding procedure between April and May 2010, were included in the study along with another group of 20 consecutively bonded patients, who had received the standard bonding protocol treatment between February and March 2010.

The inclusion criteria for selection of both groups, consisted of patients who (1) had undergone comprehensive orthodontic treatment with a completely customized lingual appliance (Incognito™, 3 M Top Service für Lingualtechnik, Bad Essen, Germany); (2) were under 18 years of age at the start of treatment; (3) had an initial and final series of intraoral photographs; (4) had an initial plaque score index of less than 15%; (5) no WSL on the lingual surfaces of the upper front teeth (canine to canine) and (6) received professional standardized dietary and oral hygiene instructions based on the German individual prophylaxis program [14], including advice on the use of fluoridated toothpaste, three times a day (1400 ppm).

Two of the 45 patients had either atypical enamel formation or palatal restorations on the evaluated tooth surfaces before commencing orthodontic treatment. Three of the 45 patients had an adjusted bonding procedure due to impacted or not fully erupted canines. These five patients were excluded from the study.

## Method

**Figure 4:**
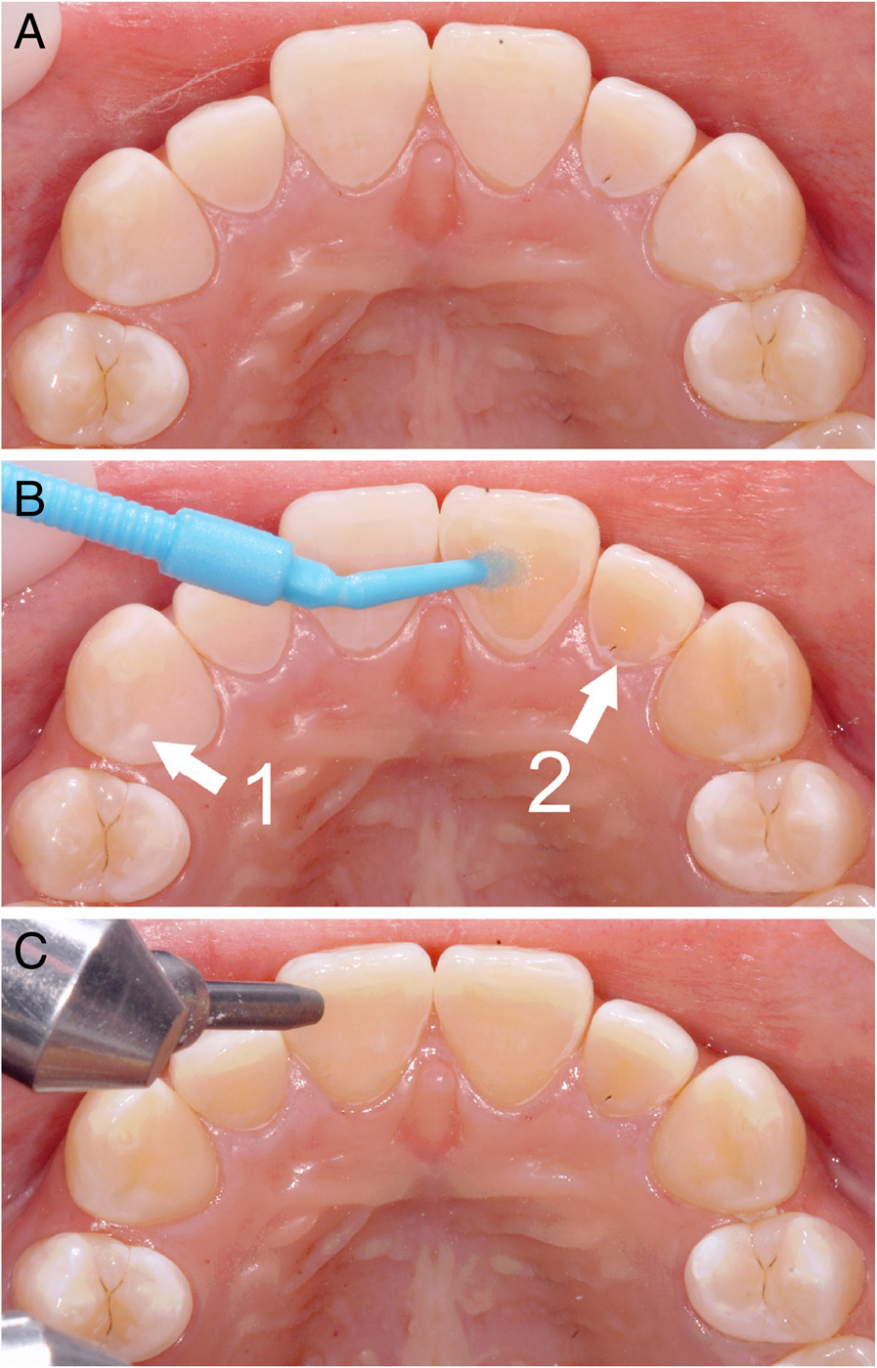
**New bonding protocol for upper anterior area. A)** Etched and air dried lingual surfaces of the maxillary anterior segment. **B)** Etched maxillary anterior lingual tooth surface (1), ExciTE® F DSC being applied to the tooth (2). **C)** Maxillary anterior lingual tooth surfaces with ExciTE® F DSC being air dried.

Of the two groups, group A (n=20) followed the standard indirect bonding protocol using the chemical cure bonding resin Maximum Cure™ (Reliance Orthodontic Products Inc., Itasca, IL, USA). This resin was placed on the individual bracket bases and the tooth surfaces prior to the insertion of the transfer tray. The patients in group B (n=20) underwent a modified bonding procedure. A supplementary layer of ExciTE® F DSC (Ivoclar Vivadent, Ellwangen, Germany), a dual cure single component enamel-dentin bonding agent, which contains fluoride (0.3% in vol.-% calcium fluoride) was applied prior to the application of Maximum Cure on the upper anterior tooth surfaces. ExciTE® F DSC has a high volume fraction of monomers, hence is less sensitive to moisture (hydrophilic). This bonding agent was placed on all lingual surfaces of the upper anterior teeth (upper right canine to upper left canine) and was air blown in order to ensure a thin layer (Figure 4).

**Figure 5:**
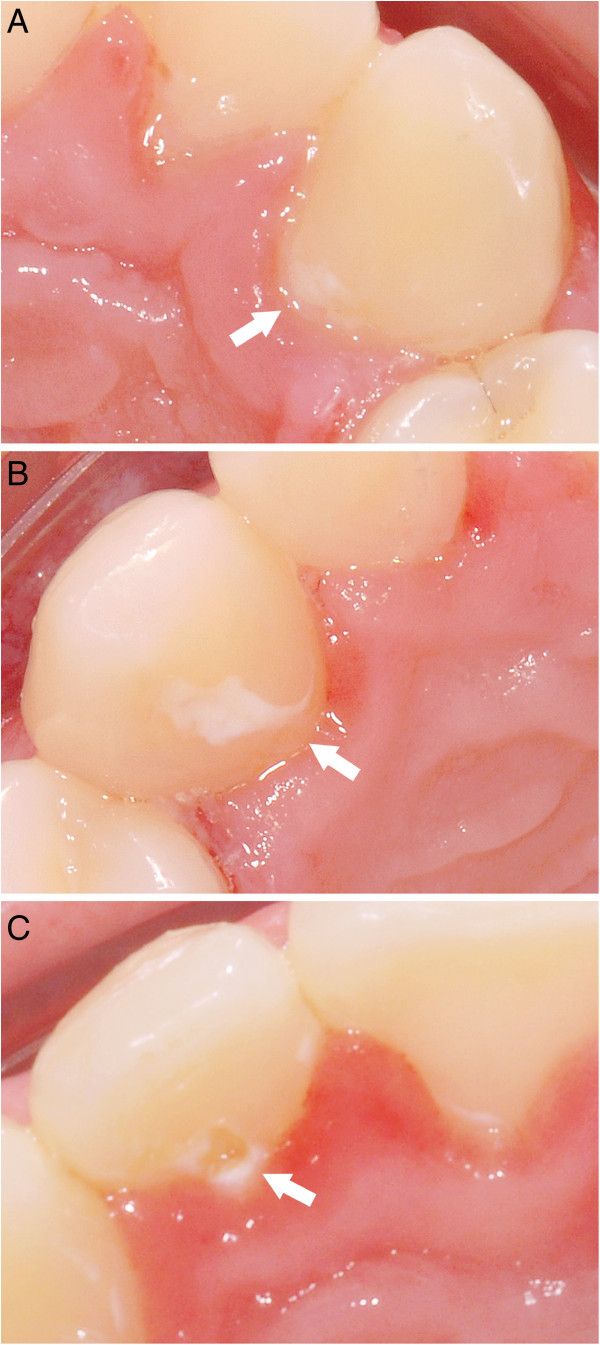
**Severity of SBLs. A)** Mild demineralization (degree I) after fixed lingual orthodontic treatment. **B)** Moderate demineralization (degree II) after fixed lingual orthodontic treatment. **C)** Severe demineralization (degree III) after fixed lingual orthodontic treatment.

The incidence of SBL were evaluated by assessing pre and post treatment photographs in a standardized manner. Pre- and post-treatment intraoral photographs of each patient were taken as part of the standard lingual orthodontic treatment, by professionals in the orthodontic clinic. A digital camera (Nikon D200, AF Mikro Nikkon 105 mm, Nikon Marco Speedlight SB-29; Nikon, Tokyo,Japan) was used. The camera was positioned at a standard distance of 40 cm, perpendicular to the maxillary incisors, for the images of the upper jaw in the occlusal plane. The intra oral mouth mirrors were rinsed with warm water and dried before taking each photograph (Figure 5A-C).

The resulting pre and post treatment images were evaluated twice, with an interval of 6 weeks apart, by three trained investigators using a computer. Maxillary canines, lateral and central incisors (UR3-UL3) were examined and the lingual tooth surfaces were scored with a binary system for the presence of sub bracket lesions (SBL). In total 240 lingual surfaces were evaluated twice by each examiner.

Patient data collected included sex, age and duration of treatment time. The latter was calculated as the complete period between the bonding of the full fixed lingual appliances and their removal. Early intervention treatment was not included in the calculation of the treatment time.

### Examiners reproducibility

The examiners were blinded for the group assignment and their previous assessment scores. In the instance of a disagreement, the tooth surface was re-examined until consensus was reached in accordance with the World Health Organization definition of acceptable consistency: that examiners should attempt to achieve at least an 80 per cent agreement between the results of duplicate examinations.

### Statistical evaluation

The statistical evaluation was carried out using SPSS® (Statistical Package for Social Sciences) for Windows 7 (SPSS, Chicago, IL, USA). The Pearson Chi-Squared test and the Fisher Exact test were used to assess significant differences between the two groups with a significance level of 5% (p < 0.05). The null hypothesis was, the addition of an extra bonding layer of an hydrophilic resin would not help to prevent SBL.

## Results

**Figure 6:**
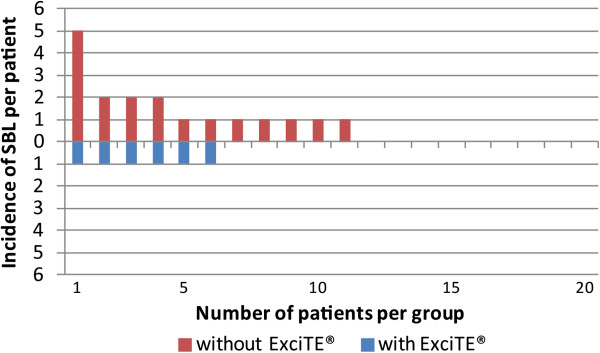
Incidence of SBL by patient and by study group.

The average age for all patients at the start of treatment was 14.0 years (range of 11.7 - 17.1 years). The overall treatment duration for the entire sample was on average 19.7 months (range of 11.5 - 27.9 months) (Figure 6). The average patient age of group A (without Excite) was 13.9 years (range 12.3 - 15.9 years) and the average treatment time was 20.8 months (range 11.5 - 27.9 months). For group B (with Excite) the average patient age at the start of treatment was 14.2 years (range 11.7 - 17.1 years) and the average treatment time was 18.7 months (range 13.3 - 25.3 months).

### Prevalence and severity

In group A, 18 SBL occurred representing 15% of the lingual tooth surfaces. In group B, SBL occurred on 6 lingual tooth surfaces (5%) (Table 1 and Figure 6). Both statistical tests showed that the difference between the two groups was statistically significant (Table 2). Therefore, the null hypothesis was rejected. The variables sex and age were not significantly related to the development of new SBL.

**Table 1:** Numbers and percentages of lingual surfaces with and without SBL development divided into group A (without ExciTE® F DSC) and group B (with ExciTE® F DSC)

				**Total number of tooth surfaces assessed**
		**SBLs development in %**	**No SBLs development in %**	
Groups	**A: without ExciTE® F DSC**	**15**	**85**	**120**
	**B: with ExciTE® F DSC**	**5**	**95**	**120**
**Total**		**10**	**90**	**240**

**Table 2:** Significance tests: Pearson Chi-squared-test and Fisher Exact-test

	**Amount**	**df**	**Exact significance (bilateral)**	**Exact significance (unilateral)**	
**Pearson Chi-squared-test**	**6,667**	**1**	**0,010**		
**Fisher exact test**				**0,016**	**0,013**
**Number of valid tooth surfaces**	**240**				

## Discussion

Comprehensive orthodontic treatment of children and adolescents has shown a lower risk of decalcification around the brackets when using fixed lingual appliances compared to labial appliances [3]. In general, decalcification on the lingual tooth surfaces was only seen in the maxillary frontal area from canine to canine [3]. Therefore, this seems to be the area at risk when using lingual appliances.

Because of the complexity of lingual orthodontic treatment, indirect bonding procedures have undisputed advantages in terms of precise bracket placement. Today, most practitioners use an indirect bonding protocol for the placement of lingual appliances [3,11,12]. This is in contrast to labial bracket bonding, which in most cases is direct bonding. Only a few orthodontists are using indirect procedures for labial bonding [15,16]. Their reasons for this are: i. shorter appointments, ii. more precise bracket placement and iii. the possibility to delegate the bonding procedure [16-18]. Surprisingly, no decalcification under the brackets (SBL) has been reported in association with labial indirect bonding procedures in children and adolescents so far [15-18]. Similar findings regarding the lingual literature can be explained by the fact that lingual orthodontic treatment was until now, nearly exclusively used in the treatment of adult patients. Only recently, with the introduction of completely customized lingual appliances (CCLA) has orthodontic treatment for younger patients become increasingly popular [2,19,20].

Two statistical tests were applied due the low number of events in one of the cells. The authors decided to compare the results of both tests and make sure that the results were not different in terms of significance. Both tests showed a statistically significant result.

As shown in this study the application of an additional bonding agent can reduce the decalcification risk on the lingual surfaces. ExciTE® F DSC may have had this effect due to: (i) the creation of an additional layer of bonding material with the reduction of porosities, (ii) easier moisture control of the enamel as it is hydrophilic, (iii) fluoride release from the material providing a protective effect [21,22]. In addition, this bonding material allows for a significantly longer working time and one therefore can check its correct application. The use of a self-cure resin requires a fast application and therefore is technically more demanding. It is important to note that ExciTE® F DSC may accelerate the polymerization of the bonding agent; for this reason the Maximum Cure was applied in the posterior segments first and in the anterior teeth secondly.

Many studies have shown the process of decalcification is fast and usually develops in the first six months of treatment, therefore especially in this period a prophylactic provision is necessary [23-26]. The average treatment time of patients in group A (without ExciTE® F DSC, following the standard bonding procedure) was 20.8 months and was longer than the average treatment time of 18.7 months in group B (with ExciTE® F DSC). The question is then if a longer treatment time of only on average 2.1 months could have an effect on the incidence of decalcification.

## Conclusion

Lingual orthodontic treatment of children and adolescents may cause decalcification under the brackets in the upper front teeth when an indirect bonding technique is applied. By adding an extra layer of a dual-cure, hydrophilic resin the incidence of these lesions can be significantly reduced. Because of the minimal severity of the SBLs in these cases, no restorative treatment was necessary.

### Clinical significance

The possibility of the correction of misaligned teeth is an important factor for restorative and aesthetic dentistry, in order to achieve a stable and healthy occlusion in the stomatognathic system. The advantages of invisible completely customized lingual appliances with reduced risk of damage to the teeth has been established and is forming an essential part of dentistry in the future.

### Ethical approval

The study was approved by the ethics committee of the Medical Faculty of the Medizinische Hochschule Hannover, Germany (No. 1189–2011). Written informed consent was obtained from all participants for data analysis and publication of the accompanying images.

## Competing interests

DW is the inventor of the Incognito-System and the founder of the former manufacturing company of Incognito which was acquired by 3 M Unitek. DW is working in private practice and not a member or associate of 3 M. All authors have no competing or financial interests in the products used.

## Authors' contributions

DW and RSP suggested the original idea for the paper. DW and FB treated the patient. FB made all medical records, made the literature search, and wrote the main part of the manuscript. RSP and DW reviewed and contributed to the writing of all iterations of the paper. All authors approved the final manuscript.

## References

[B1] Wiechmann D A new bracket system for lingual orthodontic treatment. Part 1: Theoretical background and development J Orofac Orthop 2002 63 234 245 10.1007/s00056-002-0211-5 12132311

[B2] Graber LW Vanarsdall R Vig K Orthodontics: Current Principles and Techniques 2012 18 5 Philadelphia (PA): CV Mosby 615 638

[B3] van der Veen MH Attin R Schwestka-Polly R Wiechmann D Caries outcomes after orthodontic treatment with fixed appliances: do lingual brackets make a difference? Eur J Oral Sci 2010 118 298 303 10.1111/j.1600-0722.2010.00733.x 20572865

[B4] Gorelick L Geiger AM Gwinnett AJ Incidence of white spot formation after bonding and banding Am J Orthod 1982 81 93 98 10.1016/0002-9416(82)90032-X 6758594

[B5] Mizrahi E Enamel demineralization following orthodontic treatment Am J Orthod 1982 82 62 67 10.1016/0002-9416(82)90548-6 6984291

[B6] Ogaard B Prevalence of white spot lesions in 19-year-olds: a study on untreated and orthodontically treated persons 5 years after treatment Am J Orthod Dentofacial Orthop 1989 96 423 427 10.1016/0889-5406(89)90327-2 2816842

[B7] Melrose CA Appleton J Lovius BB A scanning electron microscopic study of early enamel caries formed in vivo beneath orthodontic bands Br J Orthod 1996 23 43 47 8652497 10.1179/bjo.23.1.43

[B8] Boersma JG van der Veen MH Lagerweij MD Bokhout B Prahl-Andersen B Caries prevalence measured with QLF after treatment with fixed orthodontic appliances: influencing factors Caries Res 2005 39 41 47 10.1159/000081655 15591733

[B9] Lovrov S Hertrich K Hirschfelder U Enamel Demineralization during Fixed Orthodontic Treatment - Incidence and Correlation to Various Oral-hygiene Parameters J Orofac Orthop 2007 68 353 363 10.1007/s00056-007-0714-1 17882363

[B10] Tufekci E Dixon JS Gunsolley JC Lindauer SJ Prevalence of white spot lesions during orthodontic treatment with fixed appliances Angle Orthod 2011 81 206 210 10.2319/051710-262.1 21208070 PMC8925248

[B11] Fillion D Up-to-date lingual indirect bonding procedure J Ling Orthod 1999 1 4 8

[B12] Muller C Cuzin JF Indirektes Kleben eines individuellen lingualen Bracketsystems mit einem selbsthärtenden hydrophoben Kleber Inf Orthod Kieferorthop 2005 37 263 269 10.1055/s-2005-918223

[B13] Wiechmann D Lingual orthodontics (Part 3): Intraoral sandblasting and indirect bonding J Orofac Orthop 2000 61 280 291 10.1007/s000560050013 10961053

[B14] Social law book (Sozialgesetzbuch) Fifth Book (V) § 22: Prevention of Dental diseases (Individual prophylaxis) (§ 22:Verhütung von Zahnerkrankungen (Individualprophylaxe) Berlin, Germany: Gesetzliche Krankenversicherung [http://www.sozialgesetzbuch-sgb.de/sgbv/22.html]

[B15] Sondhi A Efficient and effective indirect bonding Am J Orthod Dentofacial Orthop 1999 115 352 359 10.1016/S0889-5406(99)70252-0 10194277

[B16] Thomas RG Indirect bonding: simplicity in action J Clin Orthod 1979 13 93 106 397232

[B17] Kothari A Indirect bonding technique World J Orthod 2006 7 389 393 17190232

[B18] Kalange JT Indirect bonding: a comprehensive review of the advantages World J Orthod 2004 5 301 307 15633375

[B19] Wiechmann D Schwestka-Polly R Pancherz H Hohoff A Control of mandibular incisors with the combined Herbst and completely customized lingual appliance–a pilot study Head Face Med 2010 6 3 10.1186/1746-160X-6-3 20219141 PMC2842253

[B20] Vu J Pancherz H Schwestka-Polly R Wiechmann D Correction of Class II, Division 2 malocclusions using a completely customized lingual appliance and the Herbst device J Orofac Orthop 2012 73 225 235 10.1007/s00056-012-0077-0 22576865

[B21] ten Cate JM Jongebloed WL Arends J Remineralization of artificial enamel lesions in vitro. IV. Influence of fluorides and diphosphonates on short- and long-term reimineralization Caries Res 1981 15 60 69 10.1159/000260501 6937252

[B22] Fischer C Lussi A Hotz P Kariostatische Wirkungsmechanismen der Fluoride Schweiz Monatsschr Zahnmed 1995 105 311 317 7716463

[B23] Balenseifen JW Madonia JV Study of dental plaque in orthodontic patients J Dent Res 1970 49 320 324 10.1177/00220345700490022101 5264596

[B24] Corbett JA Brown LR Keene HJ Horton IM Comparison of Streptococcus mutans concentrations in non-banded and banded orthodontic patients J Dent Res 1981 60 1936 1942 10.1177/00220345810600120301 6946108

[B25] Lundstrom F Krasse B Streptococcus mutans and lactobacilli frequency in orthodontic patients; the effect of chlorhexidine treatments Eur J Orthod 1987 9 109 116 3472888 10.1093/ejo/9.2.109

[B26] Rosenbloom RG Tinanoff N Salivary Streptococcus mutans levels in patients before, during, and after orthodontic treatment Am J Orthod Dentofacial Orthop 1991 100 35 37 10.1016/0889-5406(91)70046-Y 2069145

